# Widespread Presence of SPX and Its Potential Role as a Phosphorus Nutrient Regulator in Dinoflagellates

**DOI:** 10.3390/microorganisms13081867

**Published:** 2025-08-10

**Authors:** Jiashun Li, Jingtian Wang, Xiaoyu Wang, Kaidian Zhang, Senjie Lin

**Affiliations:** 1 State Key Laboratory of Marine Resource Utilization in the South China Sea, Hainan University, Haikou 570228, China; 2 State Key Laboratory of Marine Environmental Science, Xiamen University, Xiamen 361005, China; 3 Department of Marine Sciences, University of Connecticut, Groton, CT 06340, USA

**Keywords:** dinoflagellates, SPX, phosphorus limitation, homeostasis regulation

## Abstract

SPX domain-containing proteins (SPXc) are crucial for regulating phosphorus (P) homeostasis in plants. Recently, the SPX gene was identified in the diatom model *Phaeodactylum tricornutum* and shown to serve as a negative regulator of P acquisition. Whether *SPXc* occurs in dinoflagellates is unclear. Here, we report the presence and potential functions of genes encoding SPXc in dinoflagellates (*dino-SPXc*). Four classes of SPXc were identified in dinoflagellates, including the three known classes—the stand-alone SPX, SPX-EXS, and SPX-VTC—and SPX-other, with SPX and SPX-EXS being dominant. Using the *TARA Oceans* database, we investigated the taxonomic and geographic distributions of *dino-SPXc* and found variations in *dino-SPXc* expression among size classes of dinoflagellates. The harmful algal bloom-causative species *Prorocentrum shikokuense* possesses all four classes of SPXc proteins, showing a fluctuating expression pattern under different nutrient conditions and during different phases of the cell cycle and algal bloom. In addition, the *SPXc* genes in Symbiodiniaceae respond not only to P stress but also to thermal variations. These results are consistent with a role of *dino-SPXc* in maintaining P homeostasis in dinoflagellates and suggest the importance of SPX-related genes in enabling dinoflagellates to sustain population growth in nutrient-variable oceans, warranting further research.

## 1. Introduction

Phosphorus (P) is a vital nutrient for all living organisms, playing critical roles in nucleic acid synthesis, energy transfer, and cell signaling [1,2,3]. In marine ecosystems, dissolved inorganic phosphorus (DIP), primarily orthophosphate (Pi), the preferred P source for phytoplankton, is highly variable and often depleted in surface waters [4,5,6], forcing marine microorganisms to develop adaptive strategies to acquire and utilize alternative P sources. Dinoflagellates, a major group of marine phytoplankton and the main culprit of harmful algal blooms (HABs), exhibit remarkable adaptability to P-limited environments [7,8]. Their ability to thrive under varying nutrient conditions makes them ecologically versatile to thrive across the global ocean where they act as significant contributors to primary production, indispensable symbionts in coral ecosystems, and forms HAB [1]. However, the molecular mechanisms regulating P acquisition and homeostasis in dinoflagellates remain largely unexplored.

The genes encoding SPX (SYG1/PHO81/XPR1, PF03105) domain-containing proteins (*SPXc*) are known as key regulators of P homeostasis in plants and fungi [9,10,11,12,13]. These proteins are classified into subfamilies including SPX, SPX-EXS (EXS domain: PF03124), SPX-MFS (major facilitator superfamily), and SPX-RING (really interesting new gene, RING domain: PF00097) [10], which are involved in various processes such as P transport, signaling, and storage [14]. In plants, SPX proteins regulate P-starvation responses through the positive regulator phosphate starvation response protein (PHR) as an intermediate [12,15]. The SPX-EXS superfamily serves in the P-deficiency response as a Pi exporter (PHO1) and light signaling [16,17]. Members of the SPX-MFS subfamily transport phosphate into the vacuole [18].

In algae, *SPXc* have been identified only in chlorophytes and diatoms. In green algae, SPX-VTC (vacuolar transporter chaperone) and SPX-SLC (permease solute carrier 13) proteins mediate the influx and efflux of vacuolar phosphate [19,20]. A typical member of the SPX-RING subfamily in plants serves as a nitrogen limitation adaptation protein (NLA), regulating phosphate transporters in response to nitrogen (N) nutrient variations [21,22]. Recently, we identified an SPX protein in the model diatom *Phaeodactylum tricornutum* [23]. By disrupting the gene encoding it using CRISPR/Cas9 and analyzing the phenotypic change in the mutant, we demonstrated that SPX acts as a negative regulator of Pi starvation-induced genes (PSI, e.g., Pi transporter and alkaline phosphatase). Similar to plants, SPX in this diatom regulates PSI expression through PHR (also known as PSR1 in algae) as a mediator [23].

Despite their established importance in other systems, the presence, diversity, and functions of SPX homologs in dinoflagellates remain unknown. This study addresses this knowledge gap by identifying genes encoding SPX domain-containing proteins in dinoflagellates (*dino-SPXc*). Through global distribution mapping and gene expression profiling, we investigated the potential roles of *dino-SPXc* in maintaining P homeostasis. Furthermore, we examined the interplay between N and P signaling mediated by *dino-SPXc*, using the HAB-forming species *Prorocentrum shikokuense* and coral-endosymbiotic family Symbiodiniaceae as model organisms. This study broadens the taxonomic scope of a crucial P-nutrition regulator and provides new insights into the molecular and ecological strategies that dinoflagellates employ to adapt to nutrient variability in marine environments.

## 2. Materials and Methods

### 2.1. Identification and Further Analysis of dino-SPXc

We used BLAST (basic local alignment search tool) analysis to identify *SPXc* genes within dinoflagellate genomes and transcriptomes. The analysis involved querying molecular databases such as Uniprot (https://www.uniprot.org/ (accessed on 5 May 2024) and SAGER (Symbiodiniaceae and algae genomic database) [24] with SPX gene sequences from the plant model *Arabidopsis thaliana* and PFAM seed sequences (PF03105) as queries. Hits with significant sequence identity (E-value < 1 × 10^−5^) were initially identified as potential homologs of *SPXc* genes. The presence of putative SPX domains was further verified using CDD in the NCBI, Pfam, and SMART databases (E-value < 1 × 10^−5^), and sequences with SPX domains identified in at least two databases were retained for subsequent analysis. Meanwhile, the Myb_CC domain (Pfam14379) was employed as a query to potentially identify PSR1 in dinoflagellates using the SAGER database.

### 2.2. Global Expression Profiling of dino-SPXc Based on TARA Oceans Metatranscriptome Data

To assess the global expression profile of *dino-SPXc*, we examined their taxonomic and geographic distribution across data from the *Tara Oceans* project [25]. Using the SPX domain (PF03105), EXS domain (PF03124), VTC domain (PF09359), and RING domain (PF00097) as queries, we analyzed the MATOU-v1+T catalog [(Marine Atlas of TARA Oceans Unigenes + metaT (eukaryotes)] with a threshold of 1 × 10^−10^ to determine expression patterns and biogeographic distributions of *dino-SPXc* in The Ocean Gene Atlas (OGA) [26]. Samples were collected from two representative depths: surface water (SRF) and deep chlorophyll maximum (DCM) layer. Planktonic eukaryotic communities were analyzed across four size fractions: 0.8–5, 5–20, 20–180, and 180–2000 μm. Pearson correlation analysis was conducted between environmental phosphate concentration [PO_4_^3−^ (μmol/L)] and different classes of *dino-SPXc* abundance across different size fractions.

### 2.3. Expression Analysis of SPXc Genes in Prorocentrum Shikokuense and Symbiodiniaceae

Considering that bloom-forming and symbiosis capabilities are closely related to nutrient regulation, we investigated the expression of *SPXc* genes in *Prorocentrum shikokuense* and six Symbiodiniaceae species. The expression levels of *SPXc* genes in *P. shikokuense* (*Pshi-SPXc*) were analyzed using both laboratory cultures and field bloom samples. Laboratory culture transcriptomes were derived from cultures grown under different nutrient conditions, including nutrient replete (N:P = 24.5; n = 3), P-depleted (N:P = 882:1; n = 2), and N-depleted (N:P = 0.12:1; n = 3) conditions, and sampled in different phases of the light/dark cycle (n = 3), including light/dark transition at 21:00 (Cellc_21), dark period (Cellc_0 and Cellc_4), and dark/light transition at 7:00 of the next day (Cellc_7)] [27,28,29].

Field metatranscriptomes were obtained from two *P. shikokuense* blooms that occurred off Baicheng (BC) beach in Xiamen, China, and in Yangtze River Estuary (Zhejiang region), East China Sea (ECS), both in May 2014. For the BC bloom, samples were collected at 23:00 on May 6, 5:00 on May 7, and 13:00 on May 7 to cover night, morning, and day periods, respectively [30]. For the ECS bloom, a pre-bloom sample (T0) and bloom period samples (T123; the average of T1, T2, and T3) were collected on April 30 (T0), May 13 (T1), May 15 (T2), and May 20 (T3) [31]. RNA extraction and RNA-seq were carried out and reported previously [30,31]. Considering the influence of the different transcriptome sequencing depths on the samples, we normalized the expression level of *Pshi-SPXc* to that of the reference gene ubiquitin (UB). Spearman correlation analysis was conducted between *Pshi-SPXc* abundance and environmental phosphate concentration.

The expression level of *SPXc* in six Symbiodiniaceae species (from clades A, B, C, and F) under different treatments was investigated using previously reported datasets accessed from the online SAGER resource [24]. The *Symbiodinium microadriaticum* transcriptomes were from cultures grown under normal temperature (23 °C), heat stress (34 °C), heat shock (36 °C for 4 h), cold stress (16 °C for 4 h), cold shock (4 °C for 4 h), in the dark period of a 12 h:12 h light/dark cycle (sampled in the dark period), and under dark stress (6 h:18 h light/dark cycle) [32]. The transcriptomes of *Symbiodinium* sp. Y106 were from cultures grown under control conditions (25 °C, 12 h:12 h light/dark cycle), heat stress (31 °C, 12 h:12 h light/dark cycle), dark stress (25 °C, 24 h dark), and dark + heat stress (31 °C, 24 h dark) [33]. The *Breviolum minutum* transcriptome was from cultures grown at 26 °C [34]. The *Cladocopium goreaui* transcriptomes were from cultures grown in P-replete (36 μM DIP added), P-depleted (no P added), G3P (provided 36 μM glycerol phosphate), and PA (12 μM phytate provided) conditions [35]. The *Fugacium kawagutii* transcriptomes were from cultures grown under P-replete (36 μM DIP added), P-depleted (no P added), and G3P (36 μM glycerol phosphate provided) conditions [36].

## 3. Results

### 3.1. Taxonomic and Ecotypic Distribution of dino-SPXc from Genomic and Transcriptomic Databases

**Figure 1:**
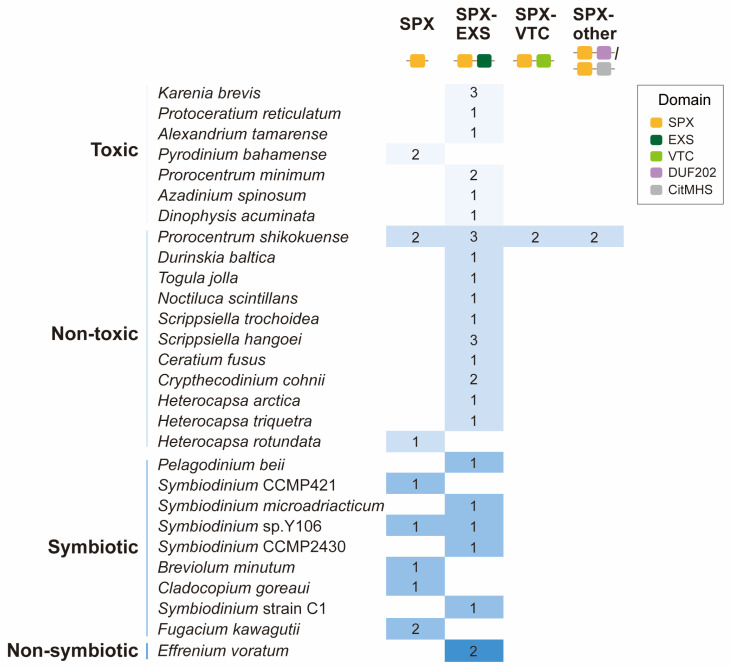
Different classes of *dino-SPXc* genes in different species. Numbers indicate the number of *SPXc* genes detected in each species.

In total, 46 *SPXc* were identified in dinoflagellates (Table S1). These SPX domain-containing dinoflagellates taxonomically encompass typical peridinin-containing lineages (arising by secondary endosymbiosis) such as *Prorocentrum*, *Alexandrium*, and *Pyrodinium* and fucoxanthin-containing (arising by tertiary endosymbiosis) taxa such as *Karenia*. They also include different ecotypes such as the kleptoplastid-containing mixotrophic *Dinophysis*, the heterotrophic *Noctiluca* and *Crypthecodinium*, and symbiotic species in the Symbiodiniaceae family. Furthermore, there are seven toxic species, 11 non-toxic species, and a non-symbiotic species (*Effrenium voratum*) from Symbiodiniaceae (Figure 1). Further sequence examination indicated that the 46 dino-SPXc proteins can be grouped into four classes, including SPX (SPX domain only), SPX-EXS (SPX and EXS domains), SPX-VTC (SPX and VTC domains), and SPX-other [SPX plus DUF202 (pfam02656) or CitMHS (pfam03600)] (Figure 1 and Table S1). Among them, the SPX-EXS class appeared most widely distributed in dinoflagellates (67% of the species examined), followed by the SPX class (24%) (Figure 1). Among the species that we examined, only *P. shikokuense* possesses SPXc proteins other than SPX and SPX-EXS, including SPX-VTC, SPX-DUF202, and SPX-CitMHS (Figure 1). As for PSR1, we did not find the existence of the Myb_CC domain in dinoflagellates, suggesting that PSR1 probably does not exist in dinoflagellates.

### 3.2. Global Distribution of dino-SPXc

**Figure 2:**
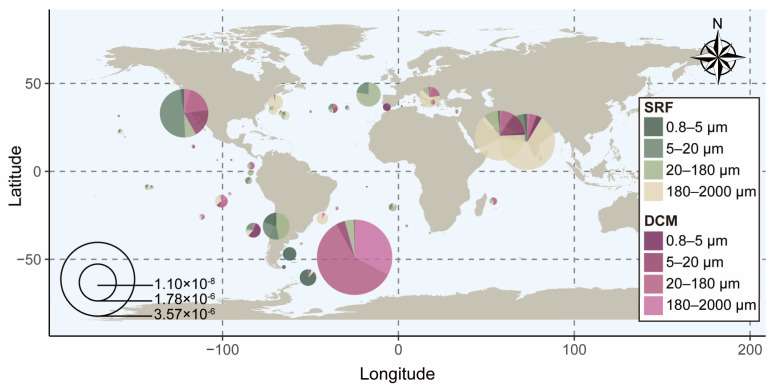
World map of the quantitative geographic distributions of dino-SPXc from the *TARA Oceans* eukaryote database in the SRF and DCM layers. The circle size represents abundance. The square color depicts the size fractions of the samples.

In the global ocean, a total of 599 significant blast hits for *SPX* genes were identified, 3% of which (18 genes) were taxonomically assigned to Dinophyceae (Table S2). These *dino-SPXc* were detected in 56 sampling sites across different size fractions and depths, and their transcript abundances normalized against ubiquitin transcript abundances (*dino-SPX*/*UB*) ranged from 1.1 × 10^−8^ to 3.57 × 10^−6^ (Figure 2 and Table S2). The expression of *dino-SPXc* in the SRF and DCM layers was detected in 52 sites and 32 sites, respectively (Table S2). Among them, *dino-SPXc* genes were expressed in both SRF and DCM layers in 28 sites, 57.1% of which showed higher expression level in the SRF layer than in the DCM layer (Figure 2). Meanwhile, biased distributions of *dino-SPXc* genes occurred in the SRF and DCM layers between size fractions (Figure 2).

In the SRF layer, *dino-SPXc* genes were predominantly expressed in the larger sized organisms, accounting for 34.6% and 40.4% of all *dino-SPXc* genes in the 20–180 μm and 180–2000 μm fractions, respectively (Figure 2). In the DCM layer, *dino-SPXc* exhibited higher expression in the smaller sized fraction, with the 5–20 μm and 20–180 μm size fractions accounting for 31.3% and 46.9% of total *dino-SPXc* expression in that layer, respectively (Figure 2).

Among the 18 *dino-SPXc* genes, four genes contained an EXS domain in addition to the SPX domain, thus belonging to the SPX-EXS class. The transcript abundance of 14 SPX genes and ambient phosphate concentration showed a significantly positive correlation in the smaller size fraction (0.8–5 μm) (*p* < 0.05), while no significant correlations were found between the other fractions and SPX-EXS genes (Table S3).

### 3.3. Expression Pattern of SPXc Genes in P. shikokuense and Symbiodiniaceae

**Figure 3:**
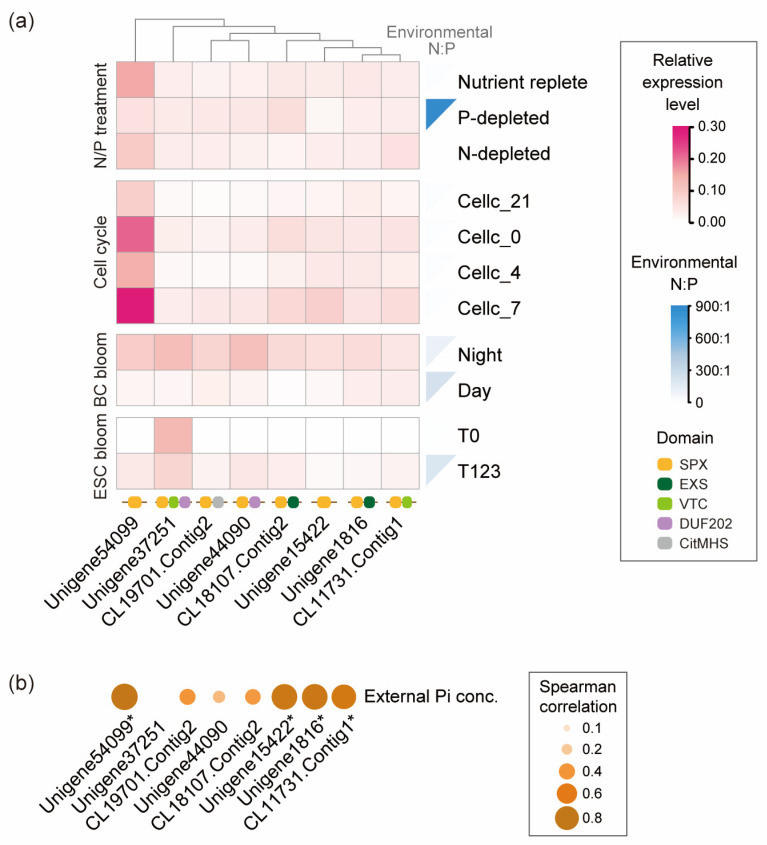
Expression pattern of *SPXc* genes in *P. shikokuense* (*Pshi-SPXc*) under different treatments and its correlations with environmental P conditions. (**a**) Expression of *Pshi-SPXc* genes under different treatments. The conditions included nutrient treatments (nutrient replete, P-depleted, and N-depleted) and the dark phase of the cell cycle (started at 21:00 and ended at 7:00 of the next day) in lab cultures, and two *P. shikokuense* blooms occurred in 2014 in China. BC bloom, *P. shikokuense* bloom in Baicheng (Xiamen region); the night and day data are from samples collected at 23:00 on 6 May and 13:00 on 7 May, respectively. ECS bloom, *P. shikokuense* bloom in East China Sea (Zhejiang region); the data are from a pre-bloom sample (T0) and bloom samples (T123). (**b**) Spearman correlation between *Pshi-SPXc* and external P level. The color bar represents the correlation index, and the asterisk after the gene name indicates a significant correlation (*p* < 0.05).

Among the dinoflagellate species covered in this study, *P. shikokuense* possesses the highest number (nine) and types (four) of *SPXc* genes (Figure 1). In the transcriptomic data from laboratory cultures and field samples, eight *Pshi-SPXc* genes showed differential expression under different treatments and environmental conditions (Figure 3). Compared with cells grown under the nutrient replete condition, two SPX genes (Unigene54099 and Unigene15422) and one SPX-EXS gene (Unigene1816) were downregulated under P-deficiency (i.e., high N:P) (Figure 3a). However, the SPX-VTC-DUF202 gene (Unigene37251) was upregulated under P-depletion (Figure 3a). Under N-depletion, two SPX-VTC genes (CL11731.Contig1 and Unigene37251) were both upregulated, while two SPX genes (Unigene54099 and Unigene15422) and two SPX-EXS genes (CL18107.Contig2 and Unigene1816) were downregulated (Figure 3a).

As for the diel pattern, the expression of *Pshi-SPXc* genes, especially Unigene54099 (SPX gene), varied throughout the dark phase (Figure 3a). All eight *Pshi-SPXc* genes exhibited lower expression at the light/dark transition (Cellc_21) and at late night (Cellc_4) than at Cellc_0 (midnight) and Cellc_7 (dark/light transition) (Figure 3a).

In the field, during the BC bloom of *P. shikokuense*, all eight *Pshi-SPXc* genes exhibited downregulation along with the increase in environmental N:P (Figure 3a). For the ESC bloom, at the T0 time point when the assemblage was dominated by diatoms, only Unigene37251 in *P. shikokuense* was detected, whereas at T123 its expression decreased, coincident with an increase in environmental N:P (Figure 3a).

When all data on gene expression and external Pi availability were polled for a general correlation analysis, all eight *Pshi-SPXc* genes showed a positive correlation with environmental Pi concentration, and four of them were significantly correlated with the external P level (Figure 3b).

The expression of *SPXc* genes in Symbiodiniaceae was found to change dramatically under different conditions (Table 1). Compared to cells grown under the P-replete condition, two SPX genes in *F. kawagutii* (Fkaw26060 and Fkaw03036) showed downregulation under P-depleted and G3P conditions (Table 1). Similarly, one SPX-EXS gene of *Symbiodinium* strain C1 (Symbiodinium-sp-C1-20140214|13393_1) was also downregulated in the P-limited and PA culture groups (Table 1). SPX in *C. goreaui* (SymbC1.scaffold4357.3) was only suppressed by G3P addition (Table 1). In addition, two SPX-EXS genes and one SPX gene in *Symbiodinium* (SmicGene7450, SymA3.s891_g10, and SymA3.s6604_g1) and one SPX gene in *C. goreaui* (SymbC1.scaffold4357.3) showed downregulation in response to heat stress (Table 1).

## 4. Discussion

### 4.1. Wide Taxonomic and Geographic Distribution of dino-SPXc

The SPXc proteins are a recognized key regulator of P homeostasis in plants and fungi [10,37]. In eukaryotic phytoplankton, *SPX* genes have been reported in chlorophytes and diatoms and shown to be widely distributed in the global ocean [23]. The findings of the present study expand the taxonomic range of algal SPXc proteins to include dinoflagellates. The study also reveals the global distribution of *dino-SPXc* and their varied expression level across different geographic regions and cell-size fractions (Figure 2).

We identified four subfamilies of SPXc proteins, SPX, SPX-EXS, SPX-VTC, and SPX-other in dinoflagellates, but most dinoflagellate species (except for *Prorocentrum*) only possess SPX and SPX-EXS proteins (Figure 1). This contrasts with diatom *P. tricornutum*, in which four subfamilies, SPX, SPX-EXS, SPX-VTC, and SPX-MFS, were identified [23]. This indicates the different evolutionary trend of *SPXc* among different algal lineages and suggests that other lineages of algae should be investigated in the future.

In plants, SPX and SPX-EXS serve in the P-deficiency response and phosphate efflux, respectively. Therefore, the wide distribution of SPX and SPX-EXS in dinoflagellate implies their significant role in cellular phosphate homeostasis regulation. Considering that SPX-VTC in other organisms is mainly involved in vacuole Pi transport for polyP synthesis, the presence of SPX-VTC in *P. shikokuense* hints on the capacity of potential P storage in polyP form under different P conditions. In support of this, a previous transcriptomic study on *P. shikokuense* under P-deficient conditions indicated the downregulation of one *Pshi-SPXc* gene involved in vacuole polyP accumulation [38]. In transcriptomes derived from *Prorocentrum cordatum*, SPX, SPX-EXS, as well as SPX-VTC were also revealed [39]. Therefore, *Prorocentrum* probably possesses diversified P regulation strategies, a potential strength of these species enabling them to form wide HABs in fluctuating P environments.

It is notable that the functional domain of PHR was identified in none of the dinoflagellates examined. In vascular plants, green algae, and diatoms, PHR proteins are known to act as an intermediate between SPX and PSI to promote P acquisition [23,40]. The absence of PHR proteins suggests a different regulatory cascade in dinoflagellates. This is striking but coincides with the distinct evolutionary process and unique molecular machinery in dinoflagellates [41].

### 4.2. Potential P Homeostasis Regulatory Mechanism in Dinoflagellate by dino-SPXc Proteins

A significant correlation was observed between the ambient DIP concentration and the expression level of SPX genes in the 0.8–5 μm size fraction but not in the >5 μm size fraction and with the expression level of SPX-EXS genes (Table S3). This suggests a predominant role of SPX in small-sized phytoplankton. This is interesting in the light of small cell size organisms with higher surface-to-volume ratios being advantageous in absorbing low-abundance nutrients [1]. A tight linkage between ambient P nutrition and *dino-SPX* in smaller sized dinoflagellate further implies that the SPX genes are potentially crucial for phytoplankton to survive in the wide P-limited ocean [42]. The lack of a significant correlation between dinoflagellate SPX-EXS genes and external Pi concentration further points to the functional differentiation of SPXc proteins, which calls for deeper research towards identifying separate categories in the future.

To further illustrate the potential SPX-mediated P homeostasis regulatory mechanism in dinoflagellates, we analyzed the expression pattern of *SPXc* genes in *Prorocentrum* and Symbiodiniaceae under various conditions (Figure 3 and Table 1). The downregulation of all four SPX proteins in *P. shikokuense* and *F. kawagutii* under P-depleted conditions (Figure 3a and Table 1) is consistent with their typical role as negative regulators of P uptake mechanisms demonstrated in the diatom *P. tricornutum* [23]. However, some SPX-EXS and SPX-VTC proteins were upregulated in P-depleted *P. shikokuense* cells (Figure 3a). Similarly, the *SPXc* genes in P-depleted *P. shikokuense* and *P. tricornutum* cells were observed to be differentially regulated under Pi deficiency [23,38,43]. As SPX-EXS and SPX-VTC serve in membrane P efflux and vacuolar phosphate influx for polyphosphate synthesis, respectively, their upregulation under P scarcity suggests the possibility that they serve different roles from regulating P uptake.

The cellular N metabolism is another important piece in nutritional balance maintenance in phytoplankton, and the cross-talk between N and P signaling has been demonstrated in the diatom *P. tricornutum* [43]. Here, the upregulation of two SPX-VTC genes in *P. shikokuense* cells grown under N-depletion (Figure 3a) implies induced vacuole Pi influx in an imbalanced N:P environment. In this case, the downregulation of SPX-EXS proteins (Figure 3a) further suggests that the apparent extra intracellular Pi under N-depleted conditions is not excreted extracellularly but used in polyP synthesis in the vacuole. If verified experimentally, this would represent a tightly regulated mechanisms of P metabolism in this dinoflagellate, potentially contributing to its ability to thrive under P-depleted environmental conditions. Furthermore, SPX, SPX-EXS, and SPX-VTC in bloom-forming *P. shikokuense* were downregulated in high-N:P water (Figure 3a), implying the lessening of SPXc proteins’ inhibitory effects on PSI to promote P uptake, downregulating Pi efflux, and reducing vacuole P storage in response to the progressively increasing environmental N:P during the bloom. The results suggest the occurrence of an interplay between N and P nutrition through *dino-SPXc*, which warrants further studies using a functional genetic approach (e.g., gene editing).

The algal cell cycle is strongly influenced by P nutrient availability [44]. In *P. shikokuense*, DNA duplication and mitosis, both having high P demands, have been shown to start at the light/dark transition and in late night, respectively [45]. In the present study, we noted the downregulation of all *Pshi-SPXc* genes at the light/dark transition (Cellc_21) and in late night (Cellc_4), implying a role of the suppressed *SPXc* expression in enabling P acquisition when cells are undergoing DNA duplication and mitosis.

Our results also suggest that *dino-SPXc* genes’ role in P nutrition regulation may be important in dinoflagellate cells responding to thermal stress. The SPX-related genes in Symbiodiniaceae clade A and C showed downregulation under heat stress (Table 1). It has been reported that increased P uptake is required to maintain the growth and photosynthesis of the symbiont during thermal stress [46]. Our observation implies that symbiotic dinoflagellate grown under elevated temperatures may suppress the expression of *SPXc* genes, enhancing P acquisition to support population growth and photosynthetic carbon fixation.

In summary, the present study revealed the wide distribution and multiple types of dino-SPXc proteins, as well as the varied expression patterns of dino-SPXc proteins under fluctuations in P nutrient, N:P ratio, and temperature conditions. Furthermore, our data suggest that *SPXc* genes may play a vital role in regulating P homeostasis in dinoflagellates. These characteristics of *SPXc* genes in HAB-forming and symbiotic dinoflagellates highlight the importance of *SPXc* genes in enabling dinoflagellates to sustain population growth in P-variable environments. These findings provide valuable insights and open new avenues for further research to unravel the functions of SPXc proteins and their underlying regulating cascades in dinoflagellates, particularly in the context of adaptation to P variability in HABs and coral reef ecosystems.

## Figures and Tables

**Table 1:** Expression of *SPXc* genes in Symbiodiniaceae under different conditions.

	Clade A ^a^			Clade B ^b^	Clade C * ^c^		Clade F ^d^	
	SmicGene7450	SymA3.s891_g10	SymA3.s6604_g1	SymbB.v1.2.009528	SymbC1.Scaffold4357.3	Symbiodinium-sp-C1- 20140214|13393_1	Fkaw26060	Fkaw03036
P-replete	-	-	-	-	32.233	25.43	14.69	15.27
P-depleted	-	-	-	-	34.13	22.6	2.52	0.93
DOP (G3P)	-	-	-	-	25.52	27.01	0	0.97
DOP (PA)	-	-	-	-	36.1	23.65	-	-
Normal temperature	22.01	18.52	9.9	12.75	15.65	-	-	-
Heat stress	14.26	21.7	9.97	-	11.57	-	-	-
Heat shock	21.25	-	-	-	-	-	-	-
Cold stress	24.38	-	-	-	-	-	-	-
Cold shock	16.22	-	-	-	-	-	-	-
Light period	22.01	18.52	9.9	-	-	-	-	-
Dark period	23.29	-	-	-	-	-	-	-
Dark stress	18.78	23.34	8.16	-	-	-	-	-
Dark + heat stress	-	22.45	10.27	-	-	-	-	-

-, No data. * The expression level of clade C is shown as FPKM, whereas that of the other clades are indicated as TPM. ^a^ Clade A data from [32,33]. ^b^ Clade B data from [34]. ^c^ Clade C data from [35]. ^d^ Clade F data from [36].

## Data Availability

The original contributions presented in this study are included in the article/Supplementary Material. Further inquiries can be directed to the corresponding authors.

## Supplementary Materials

The following supporting information can be downloaded at: https://www.mdpi.com/article/10.3390/microorganisms13081867/s1, Table S1: Information of identified *dino-SPXc* genes; Table S2: Identified *dino-SPXc* genes in Tara Oceans; Table S3: Pearson correlation analysis between *dino-SPXc* genes and environmental phosphate concentration among different size fractions.

## Abbreviations

P	phosphorus
N	nitrogen
SPX	named after SYG1/PHO81/XPR1
PHR	phosphate starvation response protein
*dino-SPXc*	genes encoding SPX domain-containing proteins in dinoflagellate
*Pshi-SPXc*	genes encoding SPX domain-containing proteins in *Prorocentrum shikokuense*
SPX-EXS	proteins containing SPX and EXS domains
SPX-VTC	proteins containing SPX and VTC domains
SPX-other	proteins containing SPX domain plus DUF202 or CitMHS domain
PSI	Pi starvation-induced genes
HAB	harmful algal bloom
CDD	conserved domain database
SMART	simple modular architecture research tool
OGA	Ocean Gene Atlas
DCM	deep chlorophyll maximum
SRF	surface water
TPM	transcript per million
SAGER	Symbiodiniceae and algal genomic resource
